# Investigations on the adhesion of new composites for restoring cervical lesions using energy dispersive X-ray analysis and scanning electron microscopy

**DOI:** 10.1038/s41598-019-46340-3

**Published:** 2019-07-08

**Authors:** Alexandra Roman, Stefan Ioan Stratul, Darian Rusu, Marius Boariu, Andrada Soanca, Robert Balazsi, Maria Suciu, Mărioara Moldovan, Adriana Elena Bulboacă

**Affiliations:** 1 Department of Periodontology, Faculty of Dental Medicine, Iuliu Haţieganu University of Medicine and Pharmacy, 15V. Babeş St., 400012 Cluj-Napoca, Romania; 2 Department of Periodontology, Faculty of Dental Medicine, Victor Babes University of Medicine and Pharmacy, 9 Bulevardul Revolutiei din 1989, 300070 Timisoara, Romania; 3 Department of Restorative Dentistry and Endodontics, Faculty of Dental Medicine, Victor Babes University of Medicine and Pharmacy, 9 Bulevardul Revolutiei din 1989, 300070 Timisoara, Romania; 4 Department of Psychology, Babes-Bolyai University, 7 Sindicatelor St, 400029 Cluj-Napoca, Romania; 5 Electron Microscopy Integrated Laboratory (LIME), National Institute for Research and Development of Isotopic and Molecular Technologies, INCDTIM, 67-103 Donath St., 400293 Cluj-Napoca, Romania; 6 Department of Molecular Biology and Biotechnologies, Faculty of Biology and Geology, Babeş-Bolyai University, 5-7 Clinicilor St., 400006 Cluj-Napoca, Romania; 7 Raluca Ripan Institute for Research in Chemistry, Babes-Bolyai University, 30 Fântânele St., 400294 Cluj-Napoca, Romania; 8 Department of Pathophysiology, 4-6 Victor Babeș St., Iuliu Haţieganu University of Medicine and Pharmacy, 15V. Babeş St., 400012 Cluj-Napoca, Romania

**Keywords:** Polymer chemistry, Composite resin

## Abstract

Restoration of noncarious cervical lesions with resin composites is one of the treatment options, but the retention of the restorations located at the crown-root junction is still a cause of clinical concern. The aim of this study was to evaluate the adhesive properties of three experimental resin composites and two commercial materials used to restore cavities prepared on extracted teeth as well as to determine the relative elemental composition of these materials. We tested the null hypothesis, which considered that the adhesive behaviours of different resin composites did not differ. The microleakage test using tracers showed that all tested materials exhibited some degree of dentinal microleakage, although they all had good dentinal adhesion. The results failed to reject the null hypothesis. The scanning electron microscopy revealed completely adapted adhesive interfaces underneath the restorations along with well-developed hybrid layers depending on the adhesive system. Energy dispersive X-ray analysis analyses showed that the restorative materials have similar chemical compositions, with some differences between the samples from the same material. The results support the implementation of experimental resins in clinical settings.

## Introduction

Modern dentistry techniques have improved human dentation with increased longevity of teeth through improved personal oral hygiene, fluoridation, and advanced restorative procedures^1^. However, performing thorough daily oral hygiene for extended periods in association with erosive aggressions, and tooth flexure will invariably wear the tooth substance away from a cement-enamel area (the neck of the tooth) and induce cervical non-carious cervical lesions (NCCLs)^2^. Gingival consequences (e.g., gingival recession) of aggressive personal oral hygiene procedures also have been described^3^. When NCCLs are associated with gingival recessions and consequent root surface exposure a combined restorative-surgical treatment is needed^4^.

If ignored, NCCLs invariably enlarge^1^. Identification of the putative etiological factors mediating NCCLs allows control of the extrinsic abrasive and erosive factors^5^. Subsequent restoration of crown-located NCCLs strengthens the tooth and prevents fracture, pulp involvement, caries, acid dissolution, further abrasion from tooth brushing; eliminates cervical hypersensitivity; improves aesthetics and gingival health; and eases oral hygiene maintenance for the patient^1^. A good adhesion of restorative materials to dental substrates is mandatory for the long term retention of fillings restoring NCCLs. Even small marginal gaps of 1–3μm width allow the penetration of cariogenic bacteria and/or pigments that lead to marginal discoloration. The infiltrating saliva eventually leads to debonding and retention loss of NCCL restorations^6^.

Resin-modified glass ionomer cements have the highest chance of survival for restoring NCCLs^7,8^. Conventional composites placed with two-step etch-and-rinse adhesives also have good outcomes for restoring NCCLs^7,9^. When restoring NCCLs with resin composites, the material adhesive properties severely impact the stability of the filling and consequently its biological effects on periodontal and dental tissues^10^. However, the adhesion of resin composites is a complex phenomenon that depends on the intrinsic adhesive capabilities of the material, the setting reaction, the type of adhesive system, the clinical application protocol^5^, moisture control^11^, and the type and quality of dental substrates^2,12^.

Conventional investigations are performed to test the adhesive properties of resin composites^13,14^. Supplemental investigations have been proposed recently to determine specific details related to the adhesion phenomenon and the material structure. Scanning electron microscopy (SEM) provides details of the adhesive interface, and energy dispersive X-ray analysis (EDX) enables a semi-quantitative evaluation of the chemical elements on the substratum surface. EDX has been used extensively in engineering and chemistry, but is rarely used in dentistry^15,16^.

Our team evaluated the solubility and cytotoxicity of three experimental resin composites^17^ that were designed by an autochthonous research institute to restore NCCLs. The aim of this study was to evaluate the adhesive properties of these experimental resin composites and two commercial materials using optical microscopy and SEM. We tested the null hypothesis, which considered that the adhesive behaviours of different resin composites and commercial materials did not differ. We also determined the relative composition of these dental materials and of their adhesive interface by performing EDX analyses.

## Results

### Microleakage test

**Figure 1:**
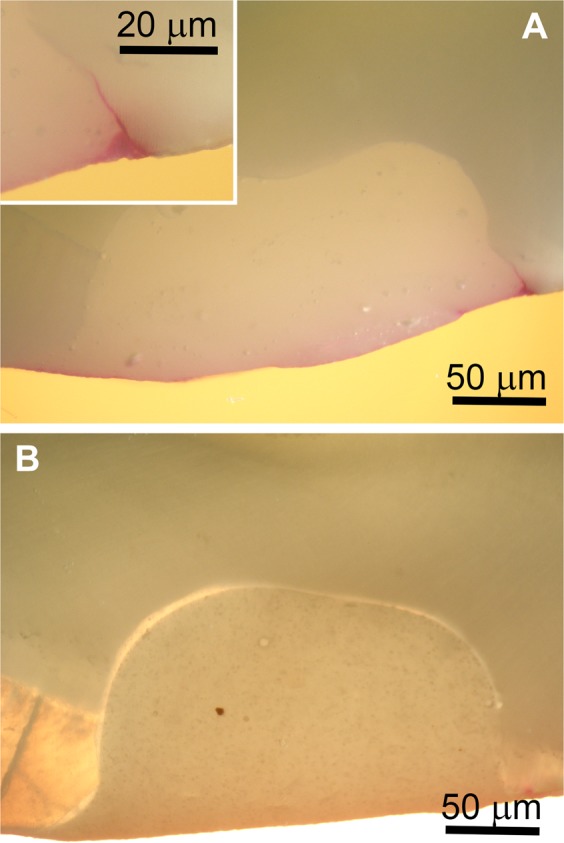
Microleakage test; optical microscopic images of two samples from a tooth restored with P2S composite. (**A**) Microleakage in dentin (40×). Inset, dye penetration (100×). (**B**) No leakage around restoration.

Cervical class V cavities were prepared on extracted teeth and restored with three experimental (P2S, P14M, and PM) and two commercial (Ge and En) resin composites. After storing in dye solution, the teeth were sectioned and the dye penetration along the restoration-tooth interface was evaluated using 17 samples for each material. Optical microscopy was used to investigate the marginal microleakage of restorations, which was highlighted as a pink infiltrating line of various lengths along the tooth-restoration interface (Fig. 1A). The ratio of marginal microleakage and total length of adhesion interface was calculated, and a value of zero was reported whenever no leakage was recorded (a very good adhesion was present). The summary of the dye penetration values for the sections of tested materials and their ratios related to the adhesive interface length are presented in Table 1.

**Table 1:** Summary of data corresponding to microleakage test according with material type.

Material	Dentin microleakage length (μm)	Adhesive interface length (μm)	Ratio of dentin microleakage length (μm): adhesive interface length (μm)
P2S			
Median (Q1 to Q3)	103 (81 to 847)	1903 (1616 to 2060)	0.059 (0.044 to 0.495)
Mean (SD)	494 (652)	1832 (305)	0.268 (0.351)
Min to max	47 to 1639	1341 to 2202	0.021 to 0.828
n	11	17	11
P14M			
Median (Q1 to Q3)	140 (121 to 164)	1977 (1905 to 2084)	0.068 (0.059 to 0.085)
Mean (SD)	156 (79)	2001 (253)	0.079 (0.043)
Min to max	64 to 331	1411 to 2389	0.032 to 0.175
n	8	17	8
PM			
Median (Q1 to Q3)	87 (85 to 89)	2080 (2062 to 2195)	0.0411 (0.0407 to 0.0415)
Mean (SD)	87 (4)	2062 (234)	0.041 (0.001)
Min to max	83 to 90	1406 to 2248	0.0403 to 0.0420
n	3	17	3
Ge			
Median (Q1 to Q3)	123 (100 to 214)	2091 (2043 to 2257)	0.055 (0.045 to 0.104)
Mean (SD)	223 (202)	2112 (269)	0.105 (0.096)
Min to max	75 to 575	1477 to 2439	0.035 to 0.272
n	9	17	9
En			
Median (Q1 to Q3)	77 (59 to 273)	2158 (1938 to 2370)	0.040 (0.030 to 0.115)
Mean (SD)	165 (151)	2165 (263)	0.075 (0.067)
Min to max	20 to 466	1832 to 2612	0.010 to 0.216
n	14	17	14

Q1 is the 25^th^ percentile; Q3 is the 75^th^ percentile; n is the number of reported samples whenever different by zero;

SD is the standard deviation.

The effect of material type (P2S, P14M, PM, Ge and En) on the ratio of dentin microleakage length (μm) to adhesive interface length (μm) was evaluated using Kruskal-Wallis ANOVA. The material type had a non-significant effect on the measured variable at the adjusted *p* < 0.01 level for the five conditions [*F*(4, 85) = 12.623, *p* = 0.0133]. This result suggests that the observed differences of the ratios of dentin microleakage length of the five material types could result from random variation. The optical micrographs revealed some degree of marginal microleakage in dentin for all tested composite resins, but no dye penetration was observed for the majority of samples (Fig. 1B). No microleakage was observed into enamel.

### Scanning electron microscopy results

**Figure 2:**
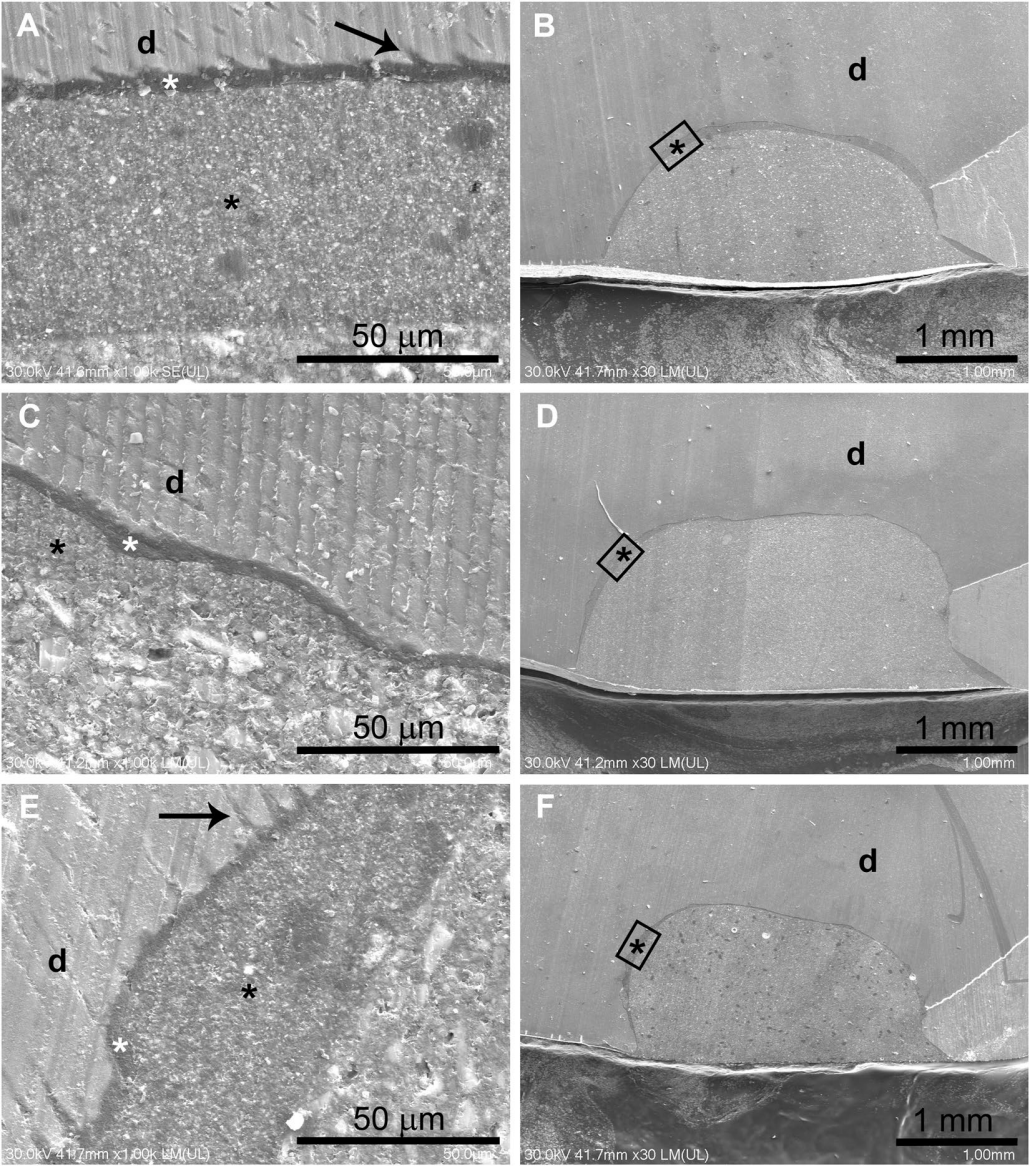
Scanning electron microscopy of experimental material restorations. (**A**) PM material structure and well-designed hybrid layer. (**B**) Continuous adhesive layer associated with PM material. (**C**) P14M material structure and adhesive interface. (**D**) Continuous adhesive layer associated with P14M material. (**E**) P2S material structure and well-designed hybrid layer. (**F**) Continuous adhesive layer associated with P2S material. Black arrow = resin tags; black asterisk = adhesive interface; white asterisk = hybrid layer; d = dentin. Label = the selected regions in the low magnification SEM images (**B**,**D**,**F**) corresponding to the magnified images (**A**,**C**,**E**).

SEM analysis showed that the PM experimental resin composite contained microfillers that were randomly distributed in the resinous matrix. A thick adhesive layer of inconstant dimensions (10–70 µm) was observed underneath the PM restorations. The granular aspect of the two-step etch-and-rinse adhesive is due to the filler content. The hybrid layer was a thin, homogenous, and more electron-dense structure of relatively uniform thickness (~2 µm) that closely followed the tooth surface and formed resinous tags ranging from 5–10 μm penetrating into the dentin (Fig. 2A). The adhesive interface underneath the P14M experimental material had a 30–90 µm thick adhesive layer, a 5 µm hybrid layer, and 2–3 µm long tags (Fig. 2C). The adhesive interfaces associated with P2S restorations had well-developed resinous tags and continuous adhesive layers: a 20–45 µm thick adhesive layer, a 5 µm hybrid layer, and tags at least 13 µm long (Fig. 2E). The hybrid filler of P14M and P2S experimental materials generally was densely packed in the resinous matrix of the restorations and had marbled appearances. Continuous adhesive interfaces were observed for all experimental materials (Fig. 2B,D,F).

**Figure 3:**
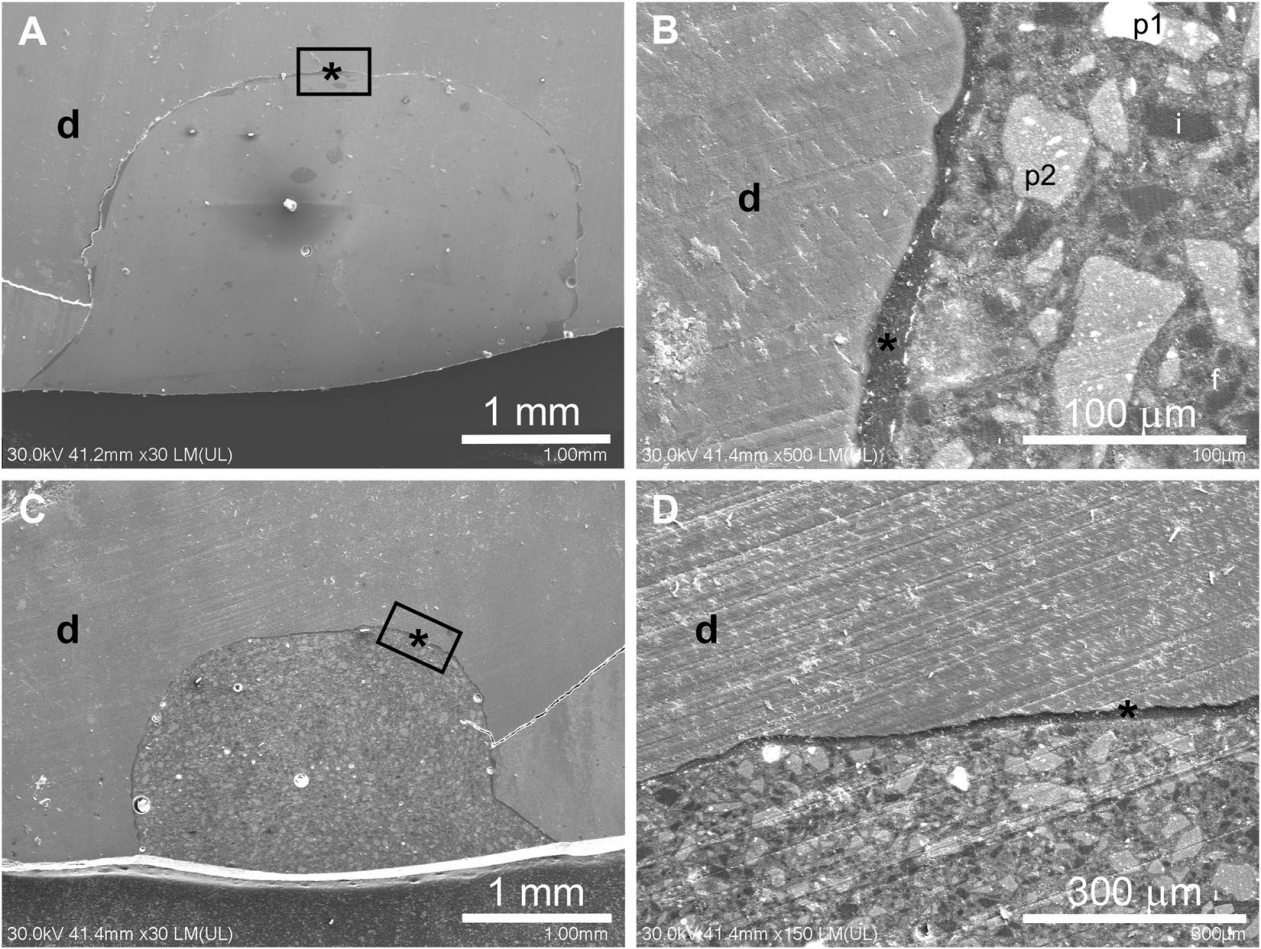
Electron microscopy of commercial resin composites. (**A**) Ge has a uniform adhesive interface (overall aspect). (**B**) Ultrastructure of Ge (prepolymerized filler silica based, *p*^2^ = prepolymerized filler containing strontium glass and lanthanoid fluoride, *i* = condensed silica particles, *f* = submicron silica particles). (**C**) En has a uniform adhesive interface (overall aspect). (**D**) Ultrastructure of En and the associated adhesive interface. Black asterisk = adhesive interface; d = dentin. Label = the selected regions in the low magnification SEM images (**A**,**C**) corresponding to the magnified images (**B**,**D**).

The uniform tooth-restoration interface associated with Ge and En suggested good adhesion and was consistent with the microleakage test results. The hybrid layer was 10–35 µm thick, with tags at least 24 µm long. SEM observations confirmed the inhomogeneous microhybrid structure of the commercial composite Ge, which was described by the manufacturer as prepolymerized filler particles (16 nm silica particles or 400 nm strontium glass and lanthanoid fluoride) of approximately 16–30 μm diameter (larger particles also are present), submicron inorganic filler (silica glass), and randomly dispersed small particles of fumed silica. SEM observations of En restorations revealed a homogenous matrix containing rarely dispersed 10–30 µm filler particles, a single homogeneous structure for the hybrid layer of ~10 µm thickness, and resinous tags at least 50 µm long expanding into the dentinal tubuli (Fig. 3).

### Energy-dispersive X-ray analysis

**Figure 4:**
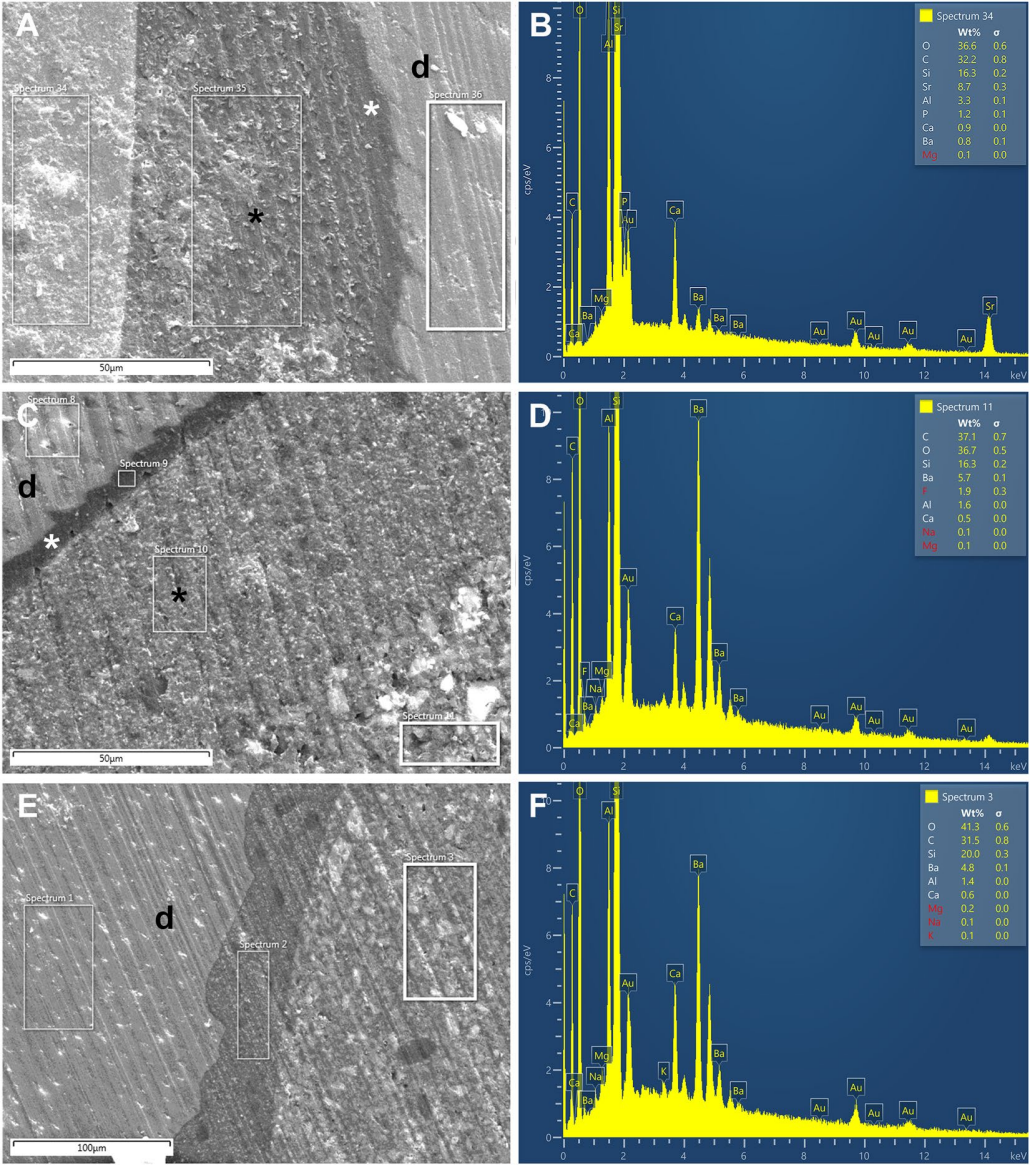
Scanning electron microscopy and EDX analysis of the samples. Restorations with PM **(A)**, P14M **(C)**, and P2S **(E)** materials and EDX results of the resin composites PM **(B)**, P14M **(D)**, and P2S **(F)**. Black asterisk = adhesive interface; white asterisk = hybrid layer; d = dentin.

**Figure 5:**
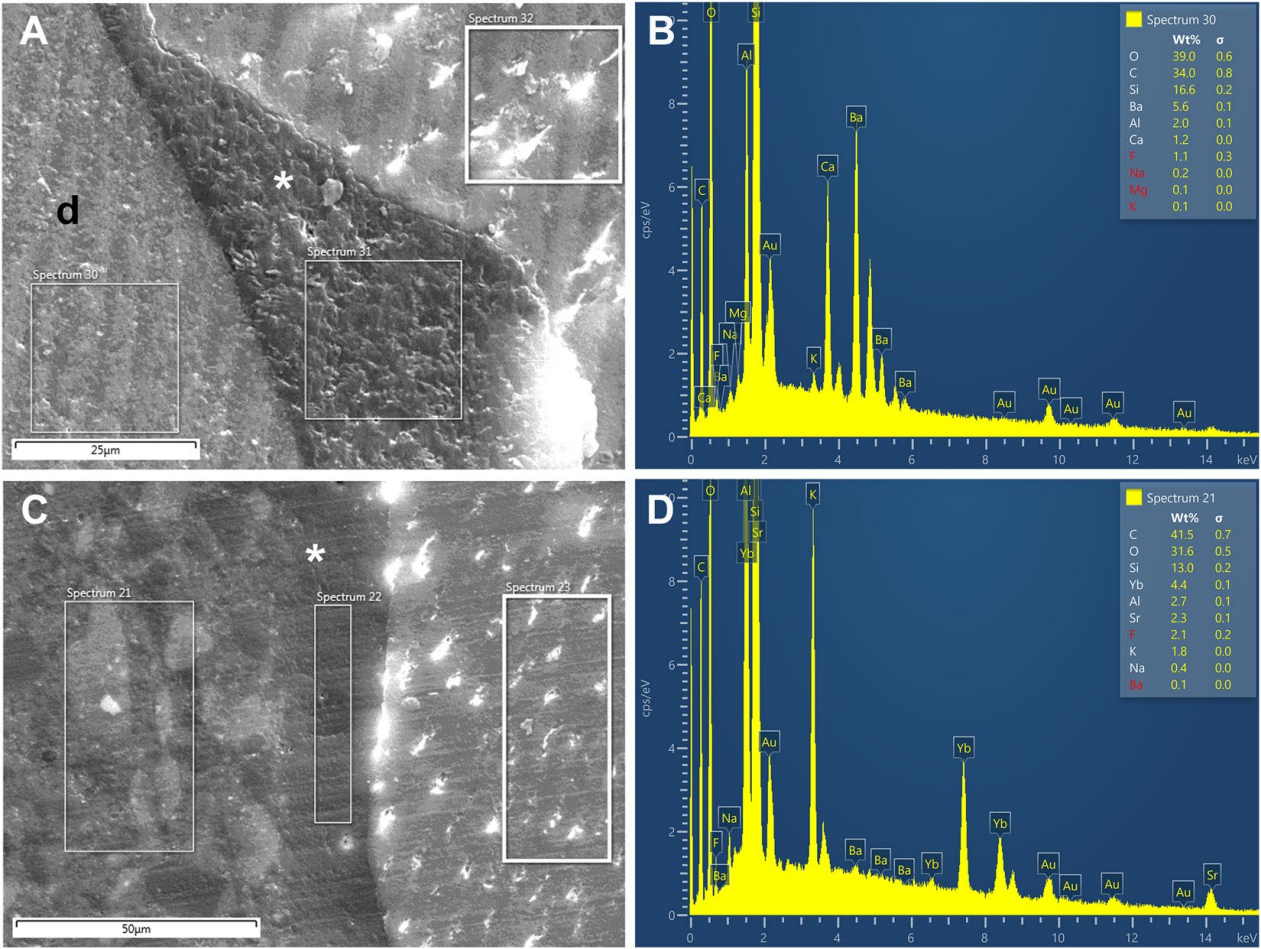
Scanning electron microscopy of samples restored with En **(A)** and Ge **(C)**, and EDX analysis of the resin composites En **(B)** and Ge **(D)**. White asterisk = hybrid layer; d = dentin.

EDX analysis was performed using 10 samples for each resin composite material. Element content was determined as percent weight (%wt) in three regions of interest (ROIs) for each sample (the material layer, adhesive layer, and dentinal area) (Figs 4 and 5).

Supplementary Table S1 presents the mean values ± SD for relative elemental content of the resin composite materials. As expected, the resin composites had different compositions. For example, silicon (Si) ranged from 18.84%wt in P2S to 12.28%wt in P14M. Barium (Ba) contents were similar in the three experimental resin composites because all contained barium oxide in the glass-based fillers. Oxygen, carbon, and silicon were the predominant elements in all composite resins. Strontium (Sr) was not detected in P14M and P2S, was detected at low levels in PM and Ge, and was detected at four times higher levels in En. Fluoride (F) had the highest content in P14M, and was not detected in En.

## Discussion

The success of resin composites for restoring NCCLs depends on adhesion to the tooth structures via adhesive systems, a conservative cavity preparation approach, and very good aesthetics^5^. Shrinkage during polymerization still remains as an important shortcoming of composite restorations, leading to bonding failure and dental and periodontal consequences^18,19^. New materials have been developed to increase the performance of restorations in cervical areas. This study investigated the adhesive capabilities of three new composite resins that were designed to restore NCCLs.

The microleakage test showed that all tested materials exhibited some degree of dentinal microleakage, although they all had comparable adhesion. The SEM micrographs of many samples revealed completely adapted adhesive interfaces underneath the restorations along with well-developed hybrid layers depending on the adhesive system. The restorative materials showed no significant differences of the adhesive performances. Therefore, these results failed to reject the null hypothesis. The data could be appreciated beyond the fact that we could not show sufficient evidence against null hypothesis. It can be observed that PM material has the best adhesion since only three samples present dentinal leakage (see Table 1) in comparison with eleven and eight samples for P2S and P14M, respectively. There are nine and fourteen samples with leakage for Ge and En, respectively. There is an important variability of the data that may be due to the differences of the intrinsic properties of dental substrates and to some errors due du material dislocation during tooth sectioning.

Our findings are consistent with those of other studies on microleakage around composite restorations^20^. The adhesive capacity of the resin composite is one factor that may influence the microleakage of directly placed composite restorations, although the substrate structure also has an important role^18,21^. NCCLs are associated with a heterogeneous hypermineralized dentin layer that resists etching^22,23^, obliteration of the majority of dentinal tubules that generates fewer resin tags^12^, and an altered composition and microstructure of the enamel and dentin consecutive to aging process^2^. From this point of view, one of the limitations of the present study was adhesion to the freshly cut dentin, which is an ideal substrate that does not generally reproduce the clinical circumstances of NCCLs.

A three-step etch-and-rinse adhesive system was used for the experimental resin composites because its efficacy is well established^24,25^ and superior to other adhesive systems^7^. A two-step self-etch adhesive and a one-step self-etch adhesive was used for the commercial resin composites, which were recommended by the manufacturers, even if they had a lower probability of being the best combination in NCCLs^7,8,26^. It seems that the combination of primer and adhesive in one product reduces the bond strength and durability of the hybrid layer^24^, thereby affecting survival of the restorations. Qualitative SEM analyses confirmed a less well-developed hybrid layer associated with one-step self-etch adhesive, although it had a well-adapted adhesive layer and good adhesion. However, the thickness of the hybrid layer is not correlated with the bond strength or the clinical performances^14^.

No enamel leakage was recorded in this study. Class V cavities are considered an advantageous research model to test the effectiveness of bonding systems^8^ because they offer no mechanical retention and are located primarily in dentin. This facilitates evaluation of the resin-dentin bond, which is less stable than the resin-enamel bond^24^. In this study, the coronal cavity bevel enhanced adhesion and “protected” the dentinal adhesive interface. Further experiments omitting bevel preparation would more rigorously test the adhesion to dentin, and would provide clinically realistic information on the adhesive capacities of the materials.

Some authors question the effectiveness of the microleakage test using tracers because a correlation between dye penetration and clinical outcomes has not been proven^14^. However, ISO standard No.11405/mentions this type of investigation, which has otherwise been used by recently published investigations^27–29^. The microleakage test using dye penetration was used in our experiment as it seemed to be the only method to evaluate new composite resin variants in the laboratory in order to gain some safety before clinical trial. The present experiment was standardized as much as possible in order to reduce the variability (a single operator for each important experimental step of the study).

EDX analyses determined the elemental compositions of three different structures; the restorative materials have similar chemical compositions, with some differences between the samples from the same material. This variation might be explained by the fact that EDX provide semi-quantitative results as well as by the technical limitations of EDX, which analyses surfaces with a maximum depth of 6 μm^30^. Some variations were related to the presence of inorganic elements in filler content of experimental materials in comparison with commercial resin composites. Barium derived from barium oxide-based glass fillers was included in experimental composites to integrate into the silica network and increase the X-ray radiopacity of the materials, which reduced the need to add a separate radiopacity agent such as ytterbium fluoride^31^. For this reason, PM and P2S materials do not contain ytterbium. Barium was also derived from barium fluoride included in P14M material in order to induce a possible cariostatic effect^32^ through fluoride release so important in cervical areas. Levels of 0.095–0.190 ppm fluoride in saliva can be sufficient for tooth decay prophylaxis^33^. Barium and strontium glasses are used to tune the refractive index of the filler particles to obtain a more transparent composite because they have a higher refractive indices than silica, which improves esthetics without altering mechanical properties^34^. Commercial composites do not contain barium. Strontium compounds were detected at low levels in Ge; ytterbium was added in this material to provide radiopacity.

Another modification of the composition of P2S and PM filler was the introduction of small quantities of hydroxyapatite - zirconium in order to make composites take advantage of the biocompatibility of hydroxyapatite and higher strength of zirconia^35^; an improved radiopacity was also targeted.

Based on the EDX analyses of material and adhesive compositions, we achieved consistent qualitative results. EDX results sustain the qualitative compositional references as provided by the manufacturers and which are responsible for the materials’ behaviour. Not only the filler composition is important but also the filler load highly influences the adhesive properties of the resin composites. High filler load produces less contraction on setting and less stress at the level of the adhesive interface^19^. The filler load is comparable for experimental and commercial materials.

The improvement of the organic component of the experimental materials was also targeted. Polycaprolactone - a biodegradable and biocompatible polymer- was introduced in PM material to enhance interfacial interaction between the polymer and the reinforcement material (filler)^36^ in order to better resist to flexural stress developed in tooth cervical areas. Unfortunately, the presence of polycaprolactone in the organic phase was probable responsible for PM instability (lower performance at sorption/solubility tests)^17^ as this molecule may be degraded through a hydrolytic mechanism by oral microorganisms^37^.

The behavior of experimental materials is the natural consequence of their composition. The present study proved comparable adhesive capacities of experimental and commercial resin composites. Previous data showed that experimental P14M and P2S composites had a good stability as proved by sorption and solubility tests; all experimental materials had a good biocompatibility in relation with oral mesenchymal stromal cells^17^. P14M and P2S materials posses properties that could be considered at least as good as the properties of commercial materials and could be manufactured at a more convenient price.

An interesting observation is related to some compositional differences detected by EDX in dentinal areas, which may be explained by the interindividual variability. The composition of apatites was reported to vary significantly^38^. In the ideal hydroxyapatite composition, some Ca ions could be substituted by similar chemical elements such as Ba or Mg, and the hydroxyl groups could be substituted by F, which modifies apatite properties^38^. This could explain the traces of these ions in dentin specimens.

The restoration of NCCLs still faces some clinical challenges^2^. Our study provides information on good adhesive behavior (under ideal *in vitro* conditions) of experimental resin composites designed to restore NCCLs. The results support the implementation of these resins in clinical settings. EDX analyses suggest that dental substrate variability is responsible in part for differences in clinical outcomes of cervical restorations.

## Methods

### Ethics

Extracted teeth were obtained with informed consent from patients according to a protocol approved by the Ethical Board of the *Iuliu Haţieganu* University (Nos 359/13.10.2014). Tooth collections were performed in accordance with relevant guidelines and regulations. Original clinical indications for tooth extractions were not related to the present study.

### Resin composites and adhesive systems

Three experimental and two commercial light-curing composite resins, shade A3, were used in this *in vitro* study to restore NCCLs (see Supplementary Table S2).

### Cavity preparation, restoration, and sample preparation

Premolars that were extracted for orthodontic reasons and free of any lesions or previous restorations were used in the present experiment. They were cleaned to remove any debris, attached soft tissues, and calculus, and stored in 4% chloramine-T until use. The teeth were used within one month after extraction^13^. A total of 55 teeth were used for the experiments, with 6 teeth used for each material for microleakage test and 5 teeth for each material used for SEM + EDX investigation.

Standard class V cavities were prepared with a mesiodistal width of 3 mm, occluso-gingival height of 3 mm, and axial depth of 1.5 mm, with the gingival margin located 1 mm below the cement-enamel junction (CEJ). The cavities were prepared on the buccal surface of teeth using diamond-coated burs (Komet S6801.314.014; Komet, Lemgo, Germany) mounted on a high speed hand-piece with air and water cooling (see Supplementary Fig. S1). Each bur was discarded after preparing five cavities. The prepared teeth were subsequently stored in distilled water as described previously^39^.

Each group of teeth was restored according to the manufacturer’s specifications, including adhesive applications. The adhesives were light-cured for 20 seconds. The cavities were restored using bulk technique, and the composite resins were light-cured for 40 seconds (Demetron A2 light-curing unit, Kerr Corporation, wavelength 450–470 nm, and light intensity of 1000 mW/cm^2^). After finishing and polishing all restorations, the restored teeth were kept in distilled water at 37 °C for 24 hours before microleakage and SEM analyses.

### Microleakage test

The root apices of the restored teeth were sealed with utility wax. The entire surface of each tooth was coated with two layers of varnish, except for a 1 mm width around the margins of the restorations. The teeth were immersed in 0.5% basic fuchsine solution for 24 hours, then rinsed thoroughly in running water for approximately 10 minutes. The teeth were sectioned in the mesiodistal direction using a low-speed diamond saw (Isomet, Buehler Ltd. Illinois, USA), resulting in three sections of 1 mm width through the middle of the restoration (see Supplementary Fig. S1). A total of 85 sections were evaluated.

Dye penetration at the gingival margin along the tooth-resin interface was evaluated with an inverted microscope (Olympus KC301, Olympus America, Inc.) at 40× magnification. Microleakage values were recorded using QuickPhoto Micro 2.2 software (Olympus, Inc). The extent of microleakage at the tooth-resin interface was quantified using a method previously developed by our research team^20,39^ as variant of a gap measurement approach previously described^40^.

Microleakage measurements (in µm) included the length of dye (pink) penetration (marked with a green line) relative to the total length of the tooth-restoration interface (marked with a yellow line). The microleakage proportion was calculated by the ratio (% µm) of the dye penetration length and the tooth-restoration interface length (see Supplementary Fig. S1).

### Scanning electron microscope (SEM) and energy-dispersive X-ray (EDX) analyses

Two dentin specimens from each tooth, which represents 10 specimens from each tested material, were prepared for SEM and EDX analyses. The same specimens were used for both analyses. The samples were dried in a graded ethanol series (50–100%), and then sputter-coated with gold. SEM was performed under high-vacuum conditions (Hitachi SU8230 STEM). Two photomicrographs were captured for each section to observe details at the tooth-restoration interface (the intimate adaptation of the adhesive-resin layer to the dentine, the hybrid layer) and the materials’ structures. The photomicrographs were analyzed by a single examiner (LB) using a blinded protocol, and measurements of the hybrid layers and resinous tags penetrating the dentin were performed using ImageJ software. EDX was performed using a Hitachi SU8230 STEM under the following conditions: 30 kV acceleration voltage, 10 µA extraction current, 15 mm working distance, Oxford Instruments EDS detector placed inside the sample chamber, and AZtec Software. Measurements were performed at the same magnifications of similar surfaces (three ROIs) for all samples: the healthy tooth, the added restorative material, and the transition material between the healthy tooth and the restorative material. Ten measurements for each material were averaged to provide a single mean value for each parameter for each specimen.

### Data analysis

The microleakage, adhesive lengths, and the ratio of dentin microleakage length (adhesive interface length) were reported as median and associated quartiles (Q1 as the 25^th^ percentile and Q3 and the 75^th^ percentile), average and standard deviation, and minimum and maximum calculated whenever measurements were different by zero. The ratios of dentin microleakage lengths (μm) were compared using Kruskal-Wallis ANOVA, with the material type (P2S, P14M, PM, Ge, and En) as independent variable. Results were considered as significant at α* = 0.05/5 = 0.01. For EDX analysis, the average concentrations of elements and standard deviations were calculated for each material.

## Supplementary information

Supplementary info

## Data Availability

The datasets generated during the current study are available from the corresponding author on reasonable request.
