# Supplementary material for: Investigations on the adhesion of new composites for restoring cervical lesions using energy dispersive X-ray analysis and scanning electron microscopy
Source: Sci Rep. 2019 Jul 8;9:9853. doi: 10.1038/s41598-019-46340-3 (PMC6614361; doi:10.1038/s41598-019-46340-3)
Supplement: Supplementary file 1 — Supplementary info [file 41598_2019_46340_MOESM1_ESM.pdf]

Title: Investigations on the adhesion of new composites for restoring cervical lesions using energy dispersive X-ray analysis and scanning electron microscopy

Authors:

Alexandra Roman, Stefan Ioan Stratul, Darian Rusu, Marius Boariu, Andrada Soanca, Robert Balazsi, Maria Suciu, Mărioara Moldovan, Adriana Elena Bulboacă

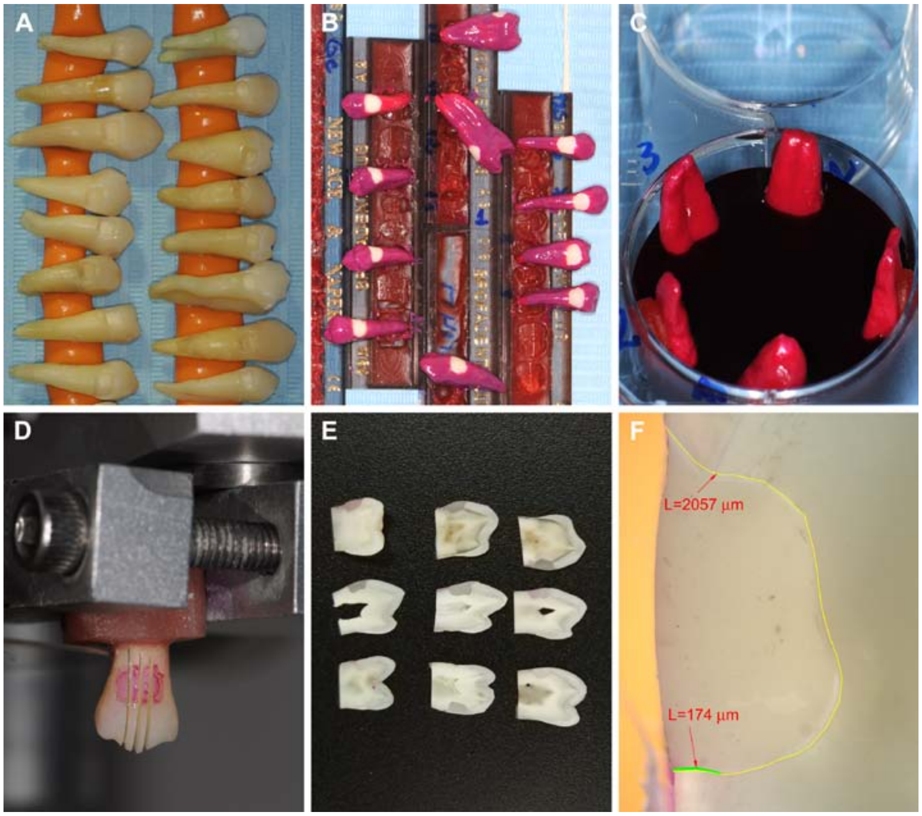

Supplementary Fig. S.1. Tooth restoration and sample preparation flowchart. **A.** Prepared class V cavities on some experimental teeth. **B.** Restored teeth coated with nail varnish. **C.** Teeth immersed in basic fuchsine solution. **D.** Tooth sectioned longitudinally with a diamond wheel saw. **E.** Sample sections for microscopic analyses. **F.** Microleakage evaluation of PM composite restoration: dye penetration is marked in green and total tooth-resin interface is marked in yellow (40× magnification).

Supplementary Table S1. Energy-dispersive X-ray analysis of elemental composition in the tested composite resin materials [percent weight (%wt)].

	PM (average ±SD)			P14M (average ±SD)			P2S (average ±SD)			En (average ±SD)			Ge (average ±SD)		
	material	Adhesive	dentine	material	adhesive	dentine	material	adhesive	dentine	material	adhesive	dentine	material	adhesive	dentine
O	39.02±1.6	29.54±1,17	39.1±0.35	32.34±5.49	26.04±4.44	40.62±4.56	36.32±5.12	26.72±2.2	38.64±0.65	39.13±2.01	23.36±2.77	39.83±0.83	32.24±1.15	29.28±4.17	37.72±1.04
C	31.4±1,8	55.22±8.37	21.62±0.45	44.9±10.31	57.1±5.85	17.3±9.83	37.3±7.4	60.08±5.14	23.12±1.48	30.23±3.18	74.73±0.97	20.8±0.4	43.36±3.24	60.64±3.79	21.42±4.61
Ca	1.2±0.17	0.2±0.07	24.2±0.29	0.4±0.12	1.06±1.33	27±4.26	0.5±0.14	0.9±1.26	24.34±0.91	0.1±0.1	1.03±1.27	24.93±0.6	0.12±0.26	2.94±0.78	26.02±2.5
P	0.24±0,53	0.08±0.17	12.94±0.35	0.08±0.17	0.66±0.93	13.48±2.48	0	0.58±0.79	12.76±0.43	0	0.46±0.45	13.33±0.3	0	1.68±0.46	13.68±1.3
Si	17.8±0,91	4.7±2.64	0	12.28±6.33	6.94±5.29	0.06±0.08	18.84±3.85	3.78±1.85	0	17.06±0.92	0.16±0.15	0	12.45±0.79	3.56±0.3	0.02±0.04
Sr	2.74±3.89	0.14±0.31	0.18±0.84	0	0	0	0	0	0	8.96±0.3	0.23±0.4	0	2.24±0.63	0	0
Al	2.44±0,49	0.3±0.41	0	1±0.93	0	0	0.76±0.7	0.04±0.08	0.04±0.08	3.73±0.2	0.03±0.05	0	2.56±0.13	0.52±0.08	0
Mg	0.14±0.11	0	0.57±0.05	0.06±0.05	0.22±0.21	0.68±0.08	0.16±0.08	0.16±0.13	0.56±0.13	0	0	0.66±0.05	0	0.58±0.22	0.52±0.14
Ba	5.38±2,63	4.88±2.75	0	5.88±1.22	4.68±1.5	0.08±0.17	4.16±0.6	3.92±2.1	0	0.73±0.05	0	0	0.04±0.08	0	0
F	0.56±0,77	1.18±0.75	0.48±0.44	1.4±0.95	0.82±0.76	0.26±0.35	0.84±0.43	0.6±0.62	0.1±0.22	0	0	0.33±0.2	1.02±0.93	0	0.12±0.13
Na	0.08±0,1	0.02±0.04	0.66±0.25	0.02±0.04	0	0.4±0.17	0	0.02±0.04	0.28±0.21	0	0	0.16±0.28	0.3±0.17	0.16±0,16	0.48±0.08
Yb	0	3.92±2.34	0	2.15±3.04	3.66±1.34	0	0	3.14±2.22	0	0	0	0	3.88±1.4	0.12±0.26	0

Supplementary Table S2. [a-e]Composition according to product brochures(c=http://cdn.gceurope.com; d=http://www.micerium.com; e=https://www.kerrdental.com). UBB-ICCRR= *Babes-Bolyai* University, *Raluca Ripan* Institute for Research in Chemistry, Cluj-Napoca Romania; Bis-GMA=- Bisphenol glycidyl dimethacrylate (UBB-ICCRR); UDMA= Urethane dimetacrylate (Sigma-Aldrich Chemie GmbH, Steinheim, Germany); HA-Zr hydroxyapatite - zirconium (UBB-ICCRR); TEGDMA= triethyleneglycol - dimethacrylate (Sigma-Aldrich); PCL-polycaprolactone diol (Sigma-Aldrich). HEMA=Hydroxyethylmethacrylate (Sigma-Aldrich).

Type of resin composite	Restorative material	Manufacturer	Matrix monomers	Filler content	Adhesive system
Hybrid	PM	UBB-ICCRR, Cluj-Napoca Romania	-Bis-GMA;<br>-UDMA;<br>-PCL diol;<br>-TEGDMA;	78 % weight, HA-Zr (particle size 0.01-60 µm and 5-8 nm); silica, glass filler (BaO) (particle size 0.1-0.35 µm);	[c]Optibond FL® (Kerr) (filled, 3-steps etch-and-rinse)<br>Etchant: 36% phosphoric acid, water<br>Primer: HEMA, ethanol<br>Adhesive: HEMA, dinatrium-hexafluorosilicate, methacrylate ester monomers and inert fillers
Hybrid	P14M	UBB-ICCRR, Cluj-Napoca Romania	-Bis-GMA;<br>-UDMA;<br>-TEGDMA;	79 % weight silica, glass filler (BaO, BaF2) (particle size 0.01-0.035 µm and 2-6 nm) ;	
Hybrid	P2S	UBB-ICCRR, Cluj-Napoca Romania	-Bis-GMA;<br>-UDMA;,,<br>-TEGDMA;	80 % weight, HA-Zr, quartz, silica;	
Nanofilled	Enamel plus HRI (En)	Micerium S.p.A, Avegno GE Italy	-[a]UDMA;<br>-Bis-GMA;<br>-1,4-butandiol-dimethacrylate;	75% weight (53% volume); glass filler: mean particle size 0.7 µm; highly dispersed silicone dioxide: mean particle size 0.04 µm;	[d]ENA-ETCH+ENA BOND®<br>(Micerium S.p.A) (2-steps self-etch adhesive)<br>Ena Etch: 37% phosphoric acid<br>Ena Bond SE primer: water, methacrylates, phosphate ester<br>Ena Bond SE Bonding: methacrylates, initiators
MFR hybrid composite	Genial anterior (Ge)	GC EUROPE N.V. Leuven	-[b]UDMA<br>-dimethacrylate<br>co-monomers;	74% weight (64% volume), Pre-polimerized fillers 16-17 µm: silica-containing, strontium and lanthanoid fluoride containing inorganic filler >100 nm: silica,<br>Inorganic filler <100 nm: fumed silica;	[e]G-ænial Bond® (GC Corporation) (filled, 1-step self-etching)<br>Phosphoric acid ester monomer, Dimethacrylate monomers, distillate water, acetone, silicon dioxid, photoinitiator
